# Correlation of the Pro-Inflammatory Cytokines IL-1β, IL-6, and TNF-α, Inflammatory Markers, and Tumor Markers with the Diagnosis and Prognosis of Colorectal Cancer

**DOI:** 10.3390/life13122261

**Published:** 2023-11-27

**Authors:** Dan Nicolae Florescu, Mihail-Virgil Boldeanu, Robert-Emmanuel Șerban, Lucian Mihai Florescu, Mircea-Sebastian Serbanescu, Mihaela Ionescu, Liliana Streba, Cristian Constantin, Cristin Constantin Vere

**Affiliations:** 1 Department of Gastroenterology, University of Medicine and Pharmacy of Craiova, 200349 Craiova, Romania; dan.florescu@umfcv.ro (D.N.F.); cristin.vere@umfcv.ro (C.C.V.); 2 Research Center of Gastroenterology and Hepatology, University of Medicine and Pharmacy of Craiova, 200638 Craiova, Romania; 3 Department of Immunology, University of Medicine and Pharmacy of Craiova, 200349 Craiova, Romania; mihail.boldeanu@umfcv.ro; 4 Department of Radiology and Medical Imaging, University of Medicine and Pharmacy of Craiova, 200349 Craiova, Romania; lucian.florescu@umfcv.ro (L.M.F.); cristian.constantin@umfcv.ro (C.C.); 5 Department of Medical Informatics and Biostatistics, University of Medicine and Pharmacy of Craiova, 200349 Craiova, Romania; mircea.serbanescu@umfcv.ro; 6 Department of Oncology, University of Medicine and Pharmacy Craiova, 2 Petru Rares Str., 200349 Craiova, Romania; liliana.streba@umfcv.ro

**Keywords:** IL-1β, IL-6, TNF-α, colorectal cancer

## Abstract

Colorectal cancer (CRC) remains one of the most important global health problems, being in the top 3 neoplasms in terms of the number of cases worldwide. Although CRC develops predominantly from the adenoma–adenocarcinoma sequence through APC gene mutations, in recent years, studies have demonstrated the role of chronic inflammation in this neoplasia pathogenesis. Cytokines are important components of chronic inflammation, being some of the host regulators in response to inflammation. The pro-inflammatory cytokines IL-1β, IL-6, and TNF-α are involved in tumor cell proliferation, angiogenesis, and metastasis and seem to strengthen each other’s mode of action, these being stimulated by the same mediators. In our study, we collected data on 68 patients with CRC and 20 healthy patients from the Gastroenterology Department of Craiova County Emergency Clinical Hospital, who were assessed between January 2022 and February 2023. The main purpose of this study was to investigate the correlation between increased plasma levels of the cytokines and the extent of the tumor, lymph nodes, and metastasis—(TNM stage), as well as the patients’ prognoses. We also compared the plasma levels of cytokines and acute inflammatory markers, namely, the erythrocyte sedimentation rate (ESR), c-reactive protein (CRP), and fibrinogen, along with the tumor markers, carcinoembryonic antigen (CEA) and carbohydrate antigen 19.9 (CA 19.9), in CRC patients. We showed that all the pro-inflammatory cytokines studied had higher levels in patients with CRC in comparison with the control group. We also showed that the acute inflammatory markers of erythrocyte sedimentation rate, C-reactive protein, and fibrinogen, and the tumor markers of CEA and CA 19.9 can be useful in diagnosis and prognosis in patients with CRC. Considering the association between pro-inflammatory cytokines and CRC, the development of new targeted therapies against IL-1β, IL-6, and TNF-α can improve patient care and the CRC survival rate.

## 1. Introduction

Approximately 2 million people were diagnosed with colorectal cancer (CRC) in 2020, with approximately 900,000 associated deaths. CRC represents a major health problem worldwide [1], despite the presence of effective screening programs that have been implemented in several countries. It is proven that in approximately 25% of cases, several types of cancer develop from chronic inflammation, which is caused by infections, chronic inflammatory diseases [2,3], or autoimmune diseases, as happens in the case of CRC with inflammatory bowel disease (IBD), gastric cancer with *Helicobacter pylori* infection [4], or hepatocellular carcinoma with hepatitis B or C viruses [5]. Asbestosis and smoking are also associated with lung cancer [6] and primary sclerosing cholangitis with cholangiocarcinoma [7]. Inflammatory cells, such as lymphocytes, neutrophils, chemokines, and cytokines are present in the tumor microenvironment, where they have different roles, such as the proliferation of tumor cells, angiogenesis, metastasis, and altering the response to chemotherapy [8,9].

Cytokines are small proteins that mediate cell communication. They adjust cell differentiation, migration, proliferation, and death [10,11]. In the tumor microenvironment, there are different levels of proinflammatory cytokines such as IL-1β, IL-6, IL-8, and TNF-α [12,13,14,15].

At the tumor sites, pro-inflammatory cytokines stimulate cell proliferation, migration, immune evasion, and angiogenesis and also reduce apoptosis [16]. IL-1 is a group of cytokines that have a proven role in the pathogenesis of numerous pathologies associated with inflammation, such as in inflammatory bowel diseases, polyarthritis rheumatoid arthritis, atherosclerosis, and asthma [17,18]. IL-6 is a pro-inflammatory cytokine that is synthesized primarily by the B and T lymphocytes and also by macrophages and monocytes, with an important role in adaptive immunity and a proven role in chronic inflammation and different types of cancer [19,20,21,22,23,24]. TNF-α is a proinflammatory cytokine that is synthesized mostly by macrophages and monocytes but also by neutrophils, fibroblasts, and T and B lymphocytes. It has a role in mediating resistance against infections, stimulating innate and adaptative immunity in chronic inflammatory diseases, and plays a role in the pathogenesis of autoimmune diseases such as rheumatoid arthritis (RA), psoriatic arthritis, and inflammatory bowel diseases [25,26,27,28].

In previous studies, it has been demonstrated that the acute phase reactants, C-reactive protein, the erythrocyte sedimentation rate (ESR), and fibrinogen levels are modified in chronic inflammation and in various types of cancers, including CRC [29,30,31,32,33,34,35]. The tumor markers of carcinoembryonic antigen (CEA) and carbohydrate antigen (CA 19.9) are not diagnostic tests and are used for monitoring patients with CRC, mostly after curative surgical interventions, and are used primarily to detect early cancer recurrence [36,37,38,39].

This study aims to compare the diagnostic and prognostic value of IL-1β, IL-6, and TNF-α in CRC, considering the connection between them and the inflammation seen in the pathogenesis of CRC [40]. This analysis is performed via MK-2 signaling, which stimulates their secretion [41] through the stimulation of the transcription factors STAT3 and NF-κB [42,43] or through reciprocal stimulation [44].

Another aim of this study was the comparison between pro-inflammatory cytokines (IL-1β, IL-6, and TNF-α), inflammatory markers (ESR, fibrinogen, and CRP), and tumor marker (CEA and CA 19.9) levels in CRC patients, alongside their correlation with the tumor characteristics (tumor extension, differentiation grade, lymph node metastasis, and distal metastasis) and the different categories of patients with this type of cancer (depending on age, gender, and lifestyle), to observe which one could be a useful biomarker in the early detection and improved prognosis of this disease.

A comparison between all pro-inflammatory cytokines and their association with the diagnosis and prognosis of CRC should be performed in future studies for an increased accuracy that would help to discover and develop new therapies in CRC.

## 2. Materials and Methods

**Figure 1:**
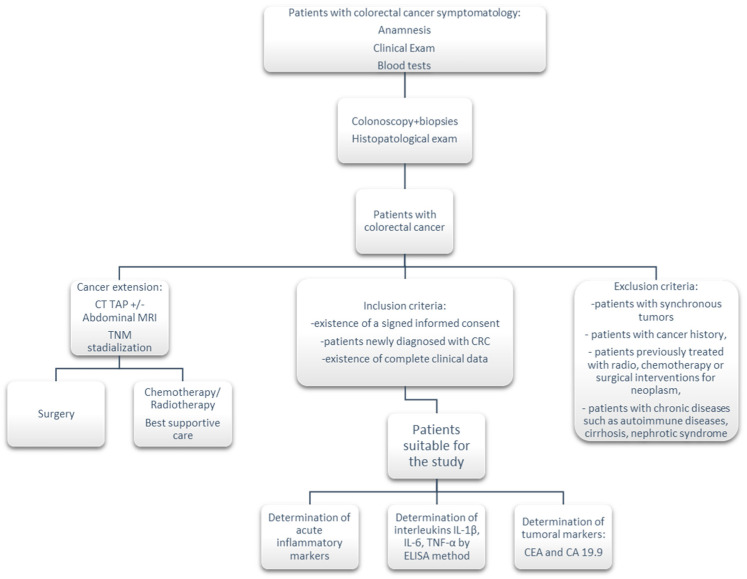
Patient selection criteria for the final study group.

This retrospective study initially included 79 patients who were diagnosed with colorectal cancer between January 2022 and February 2023 at the Craiova Emergency County Clinical Hospital and the Gastroenterology and Hepatology Research Center of the University of Medicine and Pharmacy of Craiova (Figure 1). The study was previously approved by the Ethics Committee of the University of Medicine and Pharmacy of Craiova, no. 4/21.01.2022.

### 2.1. Patients Selection

The inclusion criteria for the study were the following: the existence of a signed informed consent for data recording and processing, patients who were newly diagnosed with CRC, and the existence of complete clinical data.

The exclusion criteria specific to this study were the following: patients with synchronous tumors or with cancer history, patients previously treated with radiotherapy, chemotherapy, or surgical interventions for neoplasm, and patients with chronic diseases such as autoimmune diseases, cirrhosis, and nephrotic syndrome.

Following the application of the inclusion/exclusion criteria, 68 patients with CRC remained in this study. A control group of 20 patients (healthy patients) within the same age range was also included in the study, respecting the percentage of the male/female ratio.

After a complete anamnesis, clinical exam, and blood tests (including a hemogram and tests of liver function and renal function) the patients who were suspected of having CRC underwent an inferior digestive endoscopy, with biopsies and computed tomography or an MRI, to evaluate possible lymph node or distant metastasis; then, depending on the diagnostic stage, the patients were redirected toward surgery or oncology treatment.

The following data were also collected: patients’ clinical data, demographic characteristics, serum cytokines (IL-1β, IL-6, and TNF-α), and inflammatory marker (ESR, CRP, and fibrinogen) and tumor marker (CEA and CA 19.9) levels. Also, tests for tumors in the lymph nodes and metastasis (TNM staging) were performed based on set tumor characteristics: tumor extension (T1–T4), lymph node metastasis (N1–N2), distant metastasis (M0–M1), and differentiation degree (G1–G3).

Depending on the stage of disease at the time of diagnosis, patients were divided into patients with good prognoses (stages I and II) and patients with poor prognoses (stages III and IV).

The values of IL-1β, IL-6, and TNF-α in the serum were determined for all patients with colorectal cancer and were analyzed in relation to the TNM stage at diagnosis, along with prognosis and tumor grade, tumor characteristics (tumor location, tumor grading, tumor sizes, lymph node metastases, and metastases to distance), the demographic characteristics of the patients (urban or rural environment and level of education (level 1—secondary school degree, level 2—high school degree, level 3—university degree)), clinical data (age, sex, lifestyle—obesity, chronic alcohol consumption, or smoking), and the presence of a family history of CRC. IL-1β, IL-6, and TNF-α were also compared in relation to the acute inflammation markers of ESR, CRP, fibrinogen, and the tumor markers used in digestive cancers—CEA and CA 19.9.

### 2.2. Elisa Assays

The quantitative assessment of serum concentrations of IL-1β, IL-6, and TNF-α was achieved through the enzyme-linked immunosorbent assay (ELISA) technique, where reagents from Elabscience were used according to their usage protocol. Venous blood (5 mL) was collected à jeune, which was later centrifuged at 3000× *g* for 10 min. For the samples that were to be processed over a longer period of time, the serum sample tubes were stored at temperatures between −20 °C and −80 °C.

### 2.3. Statistical Analysis

The data collected from all patients included in the study group was initially analyzed and graphically represented using Microsoft Excel 365 (San Francisco, CA, USA). All remaining statistical analysis was performed using the Statistical Package for Social Sciences (SPSS), version 20 (IBM Corp., Armonk, NY, USA). The Shapiro-Wilk and Levene’s tests were used to assess the normality and heteroskedasticity of the continuous data. Continuous variables were presented as mean ± standard deviation (SD) and were compared using the Mann-Whitney U test or the Kruskal-Wallis H test, with a subsequent Bonferroni correction for multiple comparisons (both were used for non-Gaussian distributions). The categorical outcomes were expressed as numerical values and the corresponding percentages, and their associations were evaluated with the chi-square test or Fisher’s exact test. The statistical threshold, α, was set to 5%, and a value of *p* < 0.05 was considered statistically significant.

## 3. Results

The overall study group (both CRC patients and control patients) included 88 individuals, comprising 58 men and 30 women, with ages between 33 and 90 years old.

A Mann–Whitney U test was run to determine if there were differences in age between the two study groups. The distributions of the ages for CRC and control patients were similar, as assessed by a visual inspection. The mean age of the CRC patients was higher than the mean age of the control patients, at 68.49 ± 10.31 years old compared to 63.20 ± 10.62 years old; however, this difference between groups was not statistically significant, where U = 485.5, z = −1.939, and *p* = 0.053. Table 1 summarizes the clinical and demographic characteristics of the patients included in the study.

The demographic data presented in Table 1 indicates that only 16 patients had completed higher education (level 3); many of the patients (28) had an average level of education (level 2). Of all the patients included in the study, only 5 had a family history of CRC, and many of them (38) came from an urban environment. As for lifestyle, there were 19 patients with grade I obesity or more, 17 chronic alcohol drinkers, and 22 smokers.

**Figure 2:**
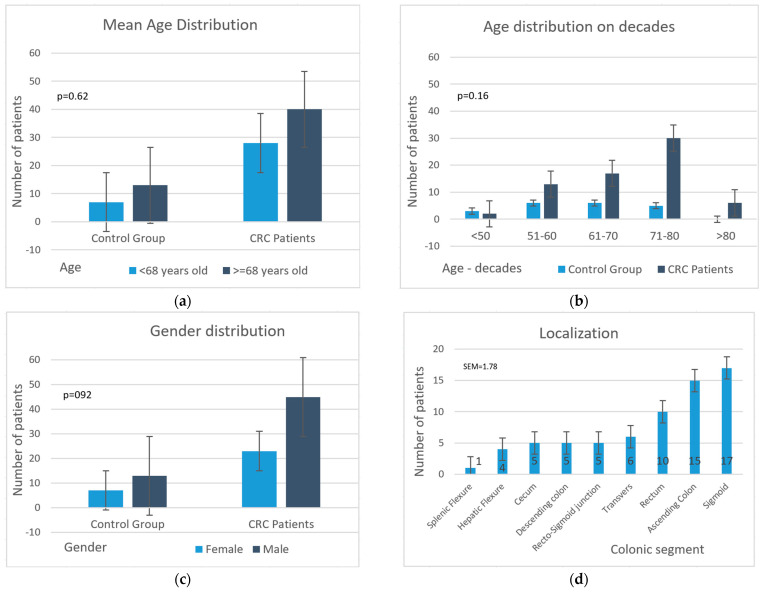
Study group-patients’ clinical data (Mann-Whitney U test): (**a**) distribution according to mean age; (**b**) age distribution according to decade; (**c**) gender; (**d**) tumor localization.

Figure 2c emphasizes the male/female ratio of the two study groups. Gender distribution was similar in both groups, with no statistically significant differences, where χ^2^(1) = 0.010, and *p* = 0.922.

The age distribution, according to decade, of the CRC patients is indicated in Figure 2b. Age was chosen as a factor when dividing the CRC patients into two distinct groups according to a threshold value, which was set at 68 years old, as indicated in Figure 2a.

Most patients had the tumor located in the sigmoid, followed by the ascending colon and rectum (Figure 2d).

### 3.1. TNM and Tumor Differentiation Classification for CRC Patients

**Figure 3:**
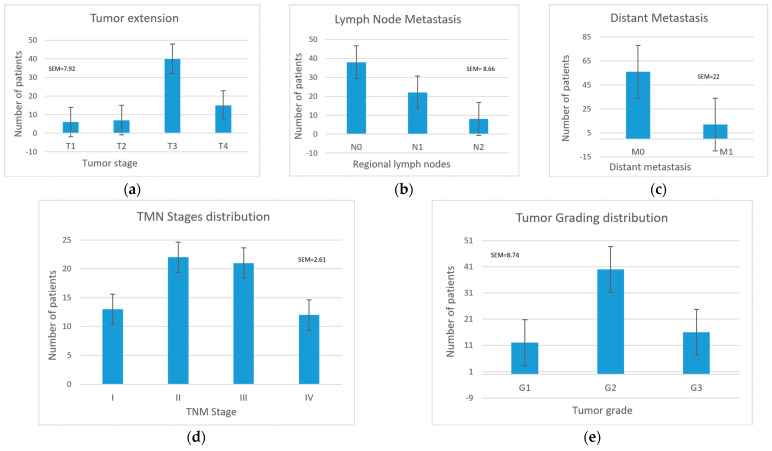
Tumoral characteristics, TNM stage, and tumor grade differentiation classification: (**a**) tumor size and extent; (**b**) lymph node metastases; (**c**) distant metastases; (**d**) TNM stage; (**e**) tumor grade.

For CRC patients, the most common tumor size and extent was T3 (40 patients, representing 58.53% of all CRC patients), followed by T4 (15 patients, 22.06%) (Figure 3a). There were no significant differences between males and females regarding the tumor size distribution (χ^2^(3) = 4.522, *p* = 0.083). A Kruskal–Wallis test was conducted to determine if there were differences in age across the T stages. The distributions of ages were similar for all groups, as assessed by a visual inspection of a boxplot. Median ages were not statistically significantly different between groups, where χ^2^(3) = 7.265, and *p* = 0.064.

Most patients had no lymph node metastasis, (38 patients; 55.88%), 22 patients showed invasion in fewer than 3 lymph nodes (N1), and only 8 patients showed invasion in more than 3 lymph nodes (N2) (Figure 3b). Only 12 patients (17.64%) had one or more distant metastases (Figure 3c).

The most frequent stage at the time of diagnosis was stage II (22 patients, 32.35%), followed by stage III (21 patients), then stages I (13 patients) and IV (12 patients). The number of patients with favorable prognoses (I and II) was equal to those with unfavorable prognoses (III and IV) (Figure 3d). In terms of tumor grading, most tumors were G2 (40 patients, 58.82%), with moderate differentiation compared to the surrounding tissue, followed by G3 tumors (16 patients) and G1 tumors (12 patients) (Figure 3e).

### 3.2. Cytokines, Inflammatory Markers, and Tumor Marker Comparisons Based on TNM Stage

**Figure 4:**
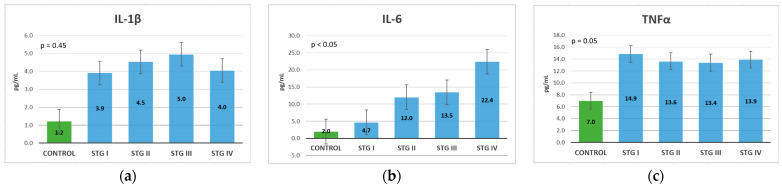
Cytokines mean values classification, based on the TNM stage (Kruskal-Wallis test): (**a**) IL-1β; (**b**) IL-6; (**c**) TNF-α.

IL-1β, IL-6, and TNF-α levels in CRC patients were increased compared to the control group, the biggest differences being recorded in the case of IL-1β. For IL-1β, an increase was observed between stages I, II, and III, but a decrease was noted in stage IV, which indicates that in the metastatic stage, this cannot be used as a prognostic marker (Figure 4a). As the stage increased, IL-6 had increasingly higher values; in stage IV, it had a level that was almost double in comparison with stage III, meaning that it could be used as a precise prognostic marker (Figure 4b). TNF-α could be used as a diagnostic marker but not so successfully as a prognostic marker, because it showed increased values in patients with CRC compared to the control group, but there were no major differences in the values between the TNM stages (Figure 4c).

**Figure 5:**
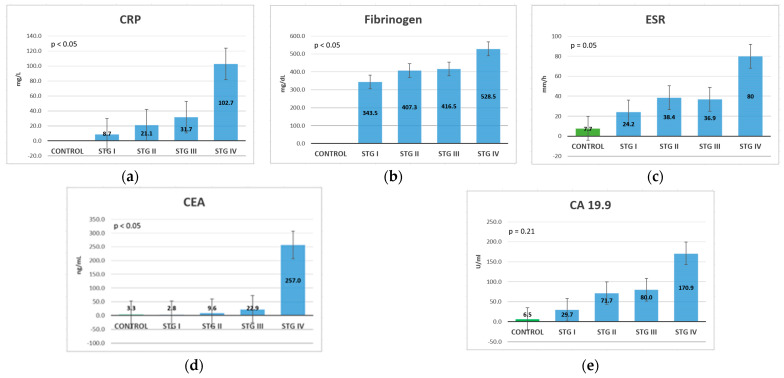
Mean values classification of the inflammatory markers and tumor markers, based on the TNM stage (Kruskal-Wallis test): (**a**) CRP (**b**) fibrinogen; (**c**) ESR; (**d**) CEA; (**e**) CA 19.9.

The inflammatory marker CRP had increasing values in more advanced stages (Figure 5a). In the control group, the ESR was within normal limits; its value increased in stage I, while in stages II and III, it showed a plateau, and in stage IV its levels increased again (Figure 5c). Fibrinogen also had higher values in more advanced stages, with an area of plateau in stages II and III (Figure 5b). Like IL-6, ESR, and CRP, fibrinogen could also be used as a CRC prognosis marker, mostly in TNM at stage IV.

The tumor marker CEA had very high levels in stage IV, with very marked differences compared to the control group and those patients with less advanced TNM stages. CEA did not exhibit markedly different levels between the control group and stage I, with a slight increase in stages II and III (Figure 5d). CA 19.9 demonstrated normal values in control group patients, a gradual increase was noted in stages I, II, and III and an important increase in stage IV (Figure 5e). This means that tumor markers could be used as negative prognostic markers in TNM at stage IV.

A Kruskal–Wallis test was conducted to determine if there were differences in cytokine levels according to tumor size and extent. The distributions of values were similar for all groups, as assessed by a visual inspection of a boxplot. For IL-1β and TNF-α, there were no statistically significant differences between groups, where *p* > 0.05. For IL-6, the values were statistically significantly different between the different T stages, where χ^2^(3) = 10.470, and *p* = 0.015. Subsequently, pairwise comparisons were performed using Dunn’s (1964) procedure. A Bonferroni correction for multiple comparisons was applied, with statistical significance accepted at the *p* < 0.0083 level. This post hoc analysis revealed statistically significant differences in IL-6 values between the T1 and T4 groups (*p* = 0.004), but not between any other group combination (Table 2).

Regarding lymph node metastasis, IL-1β and IL-6 demonstrated increasing levels with an advancement in the N stage, IL-6 demonstrating the most important differences between N stages. TNF-α did not have significantly different levels between the N stages, even a slight decrease in levels between patients without lymph node metastases (N0) and those with lymph node metastases (N1-2). Gender and age distribution were similar for N categories (χ^2^(2) = 1.708, *p* = 0.577, respectively; χ^2^(2) = 0.293 and *p* = 0.961). A Kruskal–Wallis test revealed that there were no statistically significant differences between groups, where *p* > 0.05 (Table 3).

The distributions of gender and age were similar for M categories (χ^2^(1) = 0.507, *p* = 0.477, respectively U = 418.0, z = 1.321, *p* = 0.186). A Mann-Whitney U test was run to determine if there were differences in cytokine levels between distant metastases groups. Distributions of values were not similar for all groups, as assessed by a visual inspection of a boxplot. For IL-1β and TNF-α, there were no statistically significant differences between groups, where *p* > 0.05. For IL-6, values were statistically significantly different between the different M groups, where U = 513.5, z = 2.856, and *p* = 0.004 (Table 4).

Comparing the analyzed biomarkers based on the TNM stage at diagnosis, all markers exhibited increasing levels in stage IV compared to stage I, except for TNF-α. Similarly, IL-6, CRP, and ESR exhibited gradual increases, with important differences with stage advancement. Fibrinogen also showed a gradual increase, but with slightly increased levels from one stage to another, so it cannot be considered a reliable prognostic marker. IL-1β exhibited increasing levels until stage III, then exhibited a small decrease, which means that in the metastatic stage, it cannot be used as a prognostic marker. CEA and CA 19.9 did not show substantial differences between the first three stages but exhibited a major difference between stages III and IV; thus, they can only be used as a negative prognostic marker in the TNM at stage IV (Table 5).

### 3.3. Cytokines, Inflammatory Markers, and Tumor Marker Comparisons According to the Tumor Differentiation Grades

**Figure 6:**
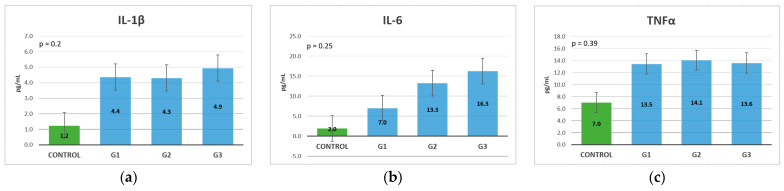
Cytokine mean values classification, based on the tumor differentiation grades: (**a**) IL-1β; (**b**) IL-6; (**c**) TNF-α.

In the tumor grading analysis, IL-1β and TNF-α did not present significantly different values in patients with G1, G2, and G3 tumors (Figure 6a,c); therefore, these cytokines cannot be taken into account in the prediction of grading in patients diagnosed with CRC. Still, they presented high levels compared to patients from the control group; therefore, these markers can be used in diagnosis but not in grading differentiation. Instead, IL-6 showed increasing values with grade progression, meaning that it is suitable for use both for diagnosis and for differentiating between the different types of tumor grades (Figure 6b).

**Figure 7:**
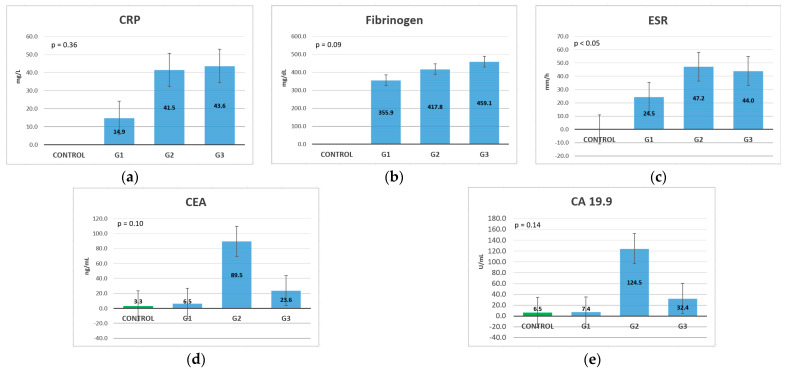
Inflammatory marker and tumor marker mean value classification, based on the tumor differentiation grade (Kruskal-Wallis test): (**a**) CRP; (**b**) fibrinogen; (**c**) ESR; (**d**) CEA; (**e**) CA 19.9.

The inflammatory marker CRP showed increased levels between G1 and G2, but there were no major differences between G2 and G3 (Figure 7a). ESR showed increasing levels between patients with G1 and G2 but showed a slight decrease in patients with G3 tumors (Figure 7c). Fibrinogen showed increasing values depending on the tumor grade (Figure 7b). Among the markers of inflammation, only fibrinogen could predict the tumor grading on each grading state, whereas CRP and ESR differentiate only G1 from the other two grades of G2 and G3, without significant differences between the last two grades.

The tumor markers CA 19.9 and CEA showed slightly increased values between patients with G1 and those in the control group but showed significantly increasing levels between patients with G1 and those with G2. Following that, the levels gradually decreased in patients with G3, which is proof of the lack of secretion of these markers in patients with undifferentiated tumors (Figure 7d,e).

In Table 6 we can see that among the studied biomarkers, IL-6 exhibited the most constant gradual increasing levels with the G stage. Fibrinogen also exhibited increasing levels along with the G stage but showed smaller differences compared to IL-6. CRP exhibited high increasing levels between G1 and G2 but showed a small difference between G2 and G3. ESR also exhibited large differences between G1 and G2 but showed a small difference with decreasing levels between G2 and G3. In the case of IL-1β, the difference was only between G2 and G3 but it was not significant. Regarding TNF-α, the G2 tumors had the highest levels, with G1 and G3 having smaller but similar levels. The tumor markers CEA and CA 19.9 also exhibited the highest levels in G2 tumors, with substantial differences in comparison with the other G stages. Unlike TNF-α, the tumor markers showed differences between G1 and G3, with higher levels for G3 tumors, but still exhibited much lower values compared to G2. 

This comparison showed us that IL-6 is the only marker associated with gradual increases according to the G stage, with an important difference in mean levels. CRP and fibrinogen are also associated with a gradual increase along with the G stage but exhibited smaller differences between the G stages compared to IL-6.

### 3.4. Analysed Markers Comparison According to Age, Gender, and Other Clinical Data

Multiple Mann–Whitney U tests were run to determine if there were differences in blood cells and cytokines between the CRC and control patients. The distributions of leucocytes, neutrophils, lymphocytes, monocytes, thrombocytes, hemoglobin, IL-1β, IL-6, and TNF-α for the two study groups were similar, as assessed by visual inspection. The statistical results are displayed in Table 7.

Neutrophils, thrombocytes, hemoglobin, and all three pro-inflammatory cytokines presented statistically significant differences between the two groups (*p* < 0.05). All parameters except lymphocytes and hemoglobin showed higher levels for CRC patients, compared to the control patients.

Age was chosen as a factor in dividing the CRC patients into two distinct groups according to a threshold value, which was set at 68 years old. A Mann–Whitney U test was applied to determine if there were differences in clinical parameters between the two groups, defined according to the age threshold, and the results are shown in Table 8.

IL-6, CRP, ESR, and CEA seemed to be influenced by the age of the patients, according to Table 8, their values being almost double for patients older than 68 years old, compared to patients younger than 68 years old. However, the biggest difference was for CA19.9, with almost tripled levels in patients ≥ 68 years old IL-1β did not exhibit major differences, but still showed slightly increased levels in patients ≥ 68 years old. TNF-α and fibrinogen showed almost equal levels between the two age groups, being slightly lower in patients ≥ 68 years old.

Table 9 shows a marker comparison according to gender. With the exception of fibrinogen, all analyzed markers showed increased levels in male patients. The biggest difference was seen for CEA, with levels being almost four times higher in male patients. There were also substantial differences in the levels of IL-6, CRP, and CA 19.9. The smallest differences between genders were for IL-1β, followed by TNF-α and ESR.

## 4. Discussion

The prognosis for patients with CRC is good in the case of a diagnosis in the early stages (I and II) and is associated with a 5-year survival rate of over 90%. In advanced stages, when patients have nodal, distant, respectively peritoneal metastasis, the prognosis is unfavorable and the survival rate at 5 years drops considerably, to below 15% (the National Cancer Institute’s Physician Data Query system).

In this study, it was shown that IL-1β, IL-6, and TNF-α are present from the early stages of colorectal cancer compared to the control group, and the levels could be used for diagnostic purposes. In TNM at stage III, both IL-6 and IL-1β have higher values, but in stage IV, only IL-6 increases, with a decrease in IL-1β in comparison with the previous stage. IL-1β and IL-6 correlate with the tumor extent and with the presence of lymph nodes. IL-6 is also associated with liver, pulmonary, and peritoneal metastasis.

All three of the studied interleukins could be used in the diagnosis of CRC, as they showed increased levels compared with the control group. For prognosis, however, the most useful marker is IL-6, because the association between increased levels and advanced stages was the most accurate of all the studied cytokines. IL-1β can also be used in the prognosis of patients with TNM stages I-II-III, but not in those with TNM at stage IV because it exhibits decreased levels when distant metastasis appears. Conversely, TNF-α has only diagnostic value; it showed almost equal levels between all stages, even with a slight decrease shown in stages II and III compared to stage I, but with a slight increase in stage IV, meaning that it cannot be used as a prognostic marker.

All inflammatory markers showed progressively increasing levels with stage progression, with the observation that fibrinogen and ESR did not show substantial differences between stages II and III, while ESR even showed a slight decrease in level in stage III compared to stage II. This means that the markers cannot differentiate among the intermediate stages, but they can be used to differentiate between a tumor limited to the submucosa and more advanced tumors, or between those associated with lymph node metastasis and those with distant metastasis. CRP showed increasing levels with every stage, the biggest difference being recorded between stages III and IV, as in the case of ESR, which means that they could be used as important prognostic markers, as with IL-6.

Regarding the tumor markers, both CEA and CA 19.9 showed increasing levels with the TNM stage progression, with very high levels and substantial differences in stage IV compared to the previous stage. CEA showed similar values between stage I patients and patients in the control group, then slightly increased in stages II and III, with greatly increased values in stage IV. We can say that in the advanced stages, it can be used as a negative prognostic marker, especially in the presence of distant metastasis. CA 19.9 showed a gradual increase, exceeding the normal value after TNM stage I. Our study indicates that both CEA and CA 19.9 exhibited higher values in more advanced stages (stage IV) in the presence of distant metastases (M1), suggesting that they are associated with an unfavorable prognosis in colorectal cancer.

CEA is the most widely used tumor marker in colorectal cancer, with high levels at diagnosis being associated with an unfavorable prognosis and low overall survival [45]. Although this marker is used after diagnosis, its importance is especially significant in post-surgical follow-ups, its increased levels being associated with recurrence and the absence of a curative effect [46,47]. CA 19.9 is also used as a tumor marker, especially in bile duct and pancreatic cancers. In colorectal cancer, CA19.9 has been associated with a worse prognosis than CEA. In this study, we have shown that it exhibits higher values in the more advanced stages (III and IV). High levels of both tumoral markers at diagnosis in colorectal cancer are related to an unfavorable prognosis [48].

Pro-inflammatory cytokines showed increased levels, emphasizing the fact that inflammation and cytokine secretion are present in tumors and that they have an important, if not decisive, role in the tumor microenvironment. IL-1β levels are high in colorectal tumor cells. The role of this cytokine in CRC is largely achieved by increasing angiogenesis and also increasing VEGF expression [49,50] through Wnt, Zeb 1, and COX 2 for inflammation, invasion, and tumor growth [51,52,53]. IL-6 has a particular role in CRC by activating the oncogenic transcription factor STAT3; this process is carried out by its binding to IL-6R, thereby playing an important pro-tumorigenic role [54,55]. At the tumor level, in colorectal cancer, TNF-α induces DNA instability and, by binding to its receptor, like IL-1β, activates the oncogenic signaling pathways, especially NF-kB and Wnt, proving the strong oncogenic role of this cytokine [56].

The therapies developed against these studied cytokines—IL-1β, IL-6, and TNF-α—can represent alternatives to current chemotherapeutic and immunological treatments, considering the toxicity and lack of effectiveness of chemotherapy in colorectal cancer. At this moment, various therapies targeting pro-inflammatory cytokines have been approved and have demonstrated a beneficial effect in other pathologies, such as: using Etanercept, Adalimumab, and Infliximab against TNF-α in inflammatory intestinal and rheumatological diseases [27,28,57]; usingTocilizumab against IL-6 in Crohn’s disease and rheumatology diseases, and also Siltuximab with Elsilimomab when directed against IL-6 in Castleman’s disease [58,59]; using Canakinumab against IL-1β in patients with cryopyrin-associated periodic syndromes [60,61]. These anti-interleukin agents also have a beneficial effect on malignancies such as prostate or lung cancer, and there are clinical trials for many other malignancies. Anti-interleukin agents may have a positive effect on colorectal cancer, considering the presence of IL-1β, IL-6, and TNF-α in this neoplasia, which was also demonstrated in this prospective study. However, more detailed studies on larger and more heterogeneous groups of patients must be performed.

As far as we know, this is the first study in which serum levels of pro-inflammatory cytokines (IL-1β, IL-6, TNF-α), inflammatory markers (ESR CRP and fibrinogen), and tumor markers (CEA and CA 19.9) have been compared in colorectal cancer.

This study has the following shortcomings: the group of patients was rather small; the group was homogenous; the patients were diagnosed in a single center, the Craiova County Emergency Clinic Hospital Research Center of Gastroenterology and Hepatology; the patients were analyzed only at admission and the results were not followed up for an analysis of overall survival and disease-free survival.

## 5. Conclusions

The presence of chronic inflammation in the pathogenesis of CRC is demonstrated in numerous studies and the current study also reinforces this idea. In addition to the inflammatory markers and tumor markers, the pro-inflammatory cytokines, IL-1β and IL-6, and TNF-α serum detection could play an important role in diagnosis and also in prognosis, although their detection incurs increased costs. They may also represent new therapeutic targets for this type of cancer, which affects many people from all over the world, but further multi-center studies involving heterogeneous groups of patients need to be performed.

## Figures and Tables

**Table 1:** Study patients—clinical and demographic characteristics.

Parameter			Number of Cases	Total
Average age (years old)	Control group	63.20 ± 10.62	20 (22.73%)	88 (100%)
	CRC patients	68.49 ± 10.31	68 (77.27%)	
Gender distribution	Control—Male		13 (65.00%)	20 (100%)
	Control—Female		7 (35.00%)	
	CRC Patients—Male		45 (66.18%)	68 (100%)
	CRC Patients—Female		23 (33.82%)	
Age distribution	Control ≥ 68 years old		13 (65.00%)	20 (100%)
	Control < 68 years old		7 (35.00%)	
	CRC Patients ≥ 68 years old		40 (58.82%)	68 (100%)
	CRC Patients < 68 years old		28 (41.18%)	
Level of education	I		24 (35.29%)	
	II		28 (41.18%)	68 (100%)
	III		16 (23.53%)	
Residence	Rural		30 (44.12%)	68 (100%)
	Urban		38 (55.88%)	
Obesity	No		49 (72.06%)	68 (100%)
	Yes		19 (27.94%)	
Smoking	No		46 (67.65%)	68 (100%)
	Yes		22 (32.35%)	
Chronic alcohol consumption	No		51 (75.00%)	68 (100%)
	Yes		17 (25.00%)	
Family history of colorectal cancer	No		63 (92.65%)	68 (100%)
	Yes		5 (7.35%)	
Tumor localization	Rectum		10 (14.71%)	68 (100%)
	Sigmoid		17 (25.00%)	
	Recto-sigmoid junction		5 (7.35%)	
	Descendent		5 (7.35%)	
	Splenic flexure		1 (1.47%)	
	Transversal		6 (8.82%)	
	Hepatic flexure		4 (5.88%)	
	Ascending		15 (22.06%)	
	Cecum		5 (7.35%)	
Tumor size and extent	T1		6 (8.82%)	68 (100%)
	T2		7 (10.29%)	
	T3		40 (58.82%)	
	T4		15 (22.06%)	
Lymph node metastases	N0		38 (55.88%)	68 (100%)
	N1		22 (32.35%)	
	N2		8 (11.76%)	
Distant metastases	M0		56 (82.35%)	68 (100%)
	M1		12 (17.65%)	
TNM stage	1		13 (19.12%)	68 (100%)
	2		22 (32.35%)	
	3		21 (30.88%)	
	4		12 (17.65%)	
Differentiation	G1		12 (17.65%)	68 (100%)
	G2		40 (58.82%)	
	G3		16 (23.53%)	

**Table 2:** Cytokine values according to tumor size and T stage.

Cytokine	T1 (N = 6) Mean	T2 (N = 7) Mean	T3 (N = 40) Mean	T4 (N = 15) Mean	*p* *
IL-1β	3.62	4.17	4.4	5.11	0.282
IL-6	3.66	5.55	5.11	12.69	0.015
TNF-α	14.45	15.2	13.78	13.13	0.060

* Kruskal–Wallis H test.

**Table 3:** Cytokine levels according to lymph node metastasis and N stage.

Cytokine	N0 (N = 38) Mean	N1 (N = 22) Mean	N2 (N = 8) Mean	*p* *
IL-1β	4.30	4.49	5.16	0.260
IL-6	10.50	14.44	20.00	0.075
TNF-α	14.10	13.35	13.96	0.077

* Kruskal-Wallis H test.

**Table 4:** Cytokine levels according to distant metastasis—M stage.

Cytokine	M0 (N = 56) Mean	M1 (N = 12) Mean	*p* *
IL-1β	4.05	4.55	0.699
IL-6	10.85	22.39	0.004
TNF-α	13.83	13.91	0.530

* Mann–Whitney U.

**Table 5:** Comparison between pro-inflammatory cytokines, inflammatory markers, and tumor markers, based on the diagnostic TNM stage.

Marker	I (N = 13) Mean	II (N = 22) Mean	III (N = 21) Mean	IV (N = 12) Mean	*p* *
CEA	2.8	9.64	22.93	256.99	0.002
CA 19.9	29.67	71.73	79.95	170.85	0.218
CRP	8.67	21.05	31.7	102.73	<0.0005
Fibrinogen	343.54	407.33	416.5	528.5	<0.0005
ESR	24.23	38.43	36.89	80.0	<0.0005
IL-1β	3.91	4.53	4.96	4.05	0.454
IL-6	4.68	12.02	13.46	22.39	0.005
TNF-α	14.85	13.62	13.41	13.91	0.050

* Kruskal–Wallis H test.

**Table 6:** Comparison between cytokines, inflammatory markers, and tumor markers, depending on the tumor differentiation grades.

Marker	G1 (N = 10) Mean	G2 (N = 39) Mean	G3 (N = 15) Mean	*p* *
CEA	6.51	89.54	23.6	0.108
CA 19.9	7.38	124.55	32.42	0.143
CRP	14.86	41.48	43.62	0.368
Fibrinogen	355.9	417.76	459.14	0.096
ESR	24.5	47.21	44.0	0.019
IL-1β	4.37	4.3	4.93	0.208
IL-6	7.04	13.28	16.31	0.251
TNF-α	13.45	14.05	13.6	0.395

* Kruskal–Wallis H test.

**Table 7:** Comparison between blood cells and pro-inflammatory cytokines, depending on the study groups (among CRC and control patients).

Interleukin	CRC Patients (N = 68) Mean	Control Patients (N = 20) Mean	*p* *
Leucocytes	8.75	7.42	0.075
Neutrophils	6.23	4.82	0.010
Lymphocytes	1.7	1.87	0.362
Monocytes	0.62	0.5	0.185
Thrombocytes	315.51	249.15	0.013
Hemoglobin	10.32	13.82	<0.0005
IL-1β	4.46	1.23	<0.0005
IL-6	12.89	1.96	<0.0005
TNF-α	13.84	7	<0.0005

* Mann–Whitney U.

**Table 8:** Comparison between cytokines, inflammatory markers, and tumor markers, depending on the age at diagnosis.

Marker	≥68 Years Old (N = 40) Mean	<68 Years Old (N = 28) Mean	*p* *
CEA	72.05	40.61	0.838
CA 19.9	132.35	50.73	0.769
CRP	46.49	26.21	0.147
Fibrinogen	416.19	418.19	0.942
ESR	50.95	29.5	0.004
IL-1β	5.02	4.07	0.127
IL-6	16.29	8.03	0.003
TNF-α	13.73	14.0	0.393

* Mann–Whitney U.

**Table 9:** Comparison between cytokines, inflammatory markers, and tumor markers, depending on gender.

Interleukin	Males (N = 68) Mean	Females (N = 20) Mean	*p* *
CEA	80.93	19.3	0.812
CA 19.9	93.61	68.42	0.174
CRP	47.78	20.45	0.071
Fibrinogen	413.85	424.88	0.714
ESR	44.21	40.41	0.938
IL-1β	4.63	4.37	0.492
IL-6	15.31	8.15	0.108
TNF-α	14.17	13.21	0.146

* Mann–Whitney U.

## Data Availability

Data are contained within the article.
